# Supplementary material for: Nanomedicine-Based Gene Delivery for a Truncated Tumor Suppressor RB94 Promotes Lung Cancer Immunity
Source: Cancers (Basel). 2022 Oct 18;14(20):5092. doi: 10.3390/cancers14205092 (PMC9600612; doi:10.3390/cancers14205092)
Supplement: Supplementary file 1 [file cancers-14-05092-s001.zip › cancers-1969511-supplementary.pdf]

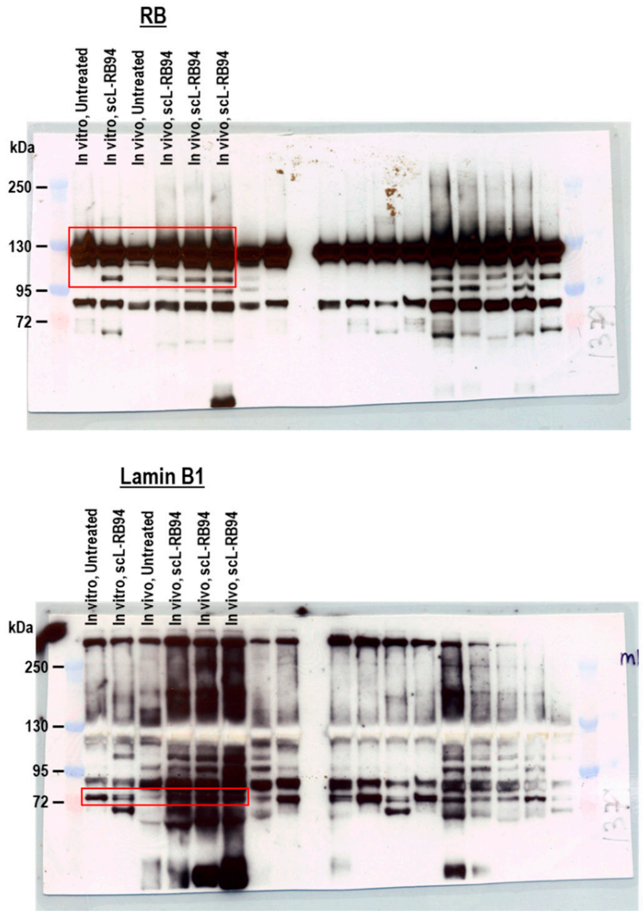

Target	Sample ID	Pixel density
RB110	In vitro, Untreated	144421
	In vitro, scL-RB94	141603
	In vivo, Untreated	117423
	In vivo, scL-RB94	155743
	In vivo, scL-RB94	157834
	In vivo, scL-RB94	163771
RB94	In vitro, Untreated	1611
	In vitro, scL-RB94	30315
	In vivo, Untreated	1842
	In vivo, scL-RB94	23429
	In vivo, scL-RB94	30400
	In vivo, scL-RB94	38304
Lamin B1	In vitro, Untreated	29317
	In vitro, scL-RB94	24538
	In vivo, Untreated	15348
	In vivo, scL-RB94	64211
	In vivo, scL-RB94	64845
	In vivo, scL-RB94	64416

Figure S1. Original whole Western blot Images for Figure 1A. The densitometry readings for the western bands are shown in table.

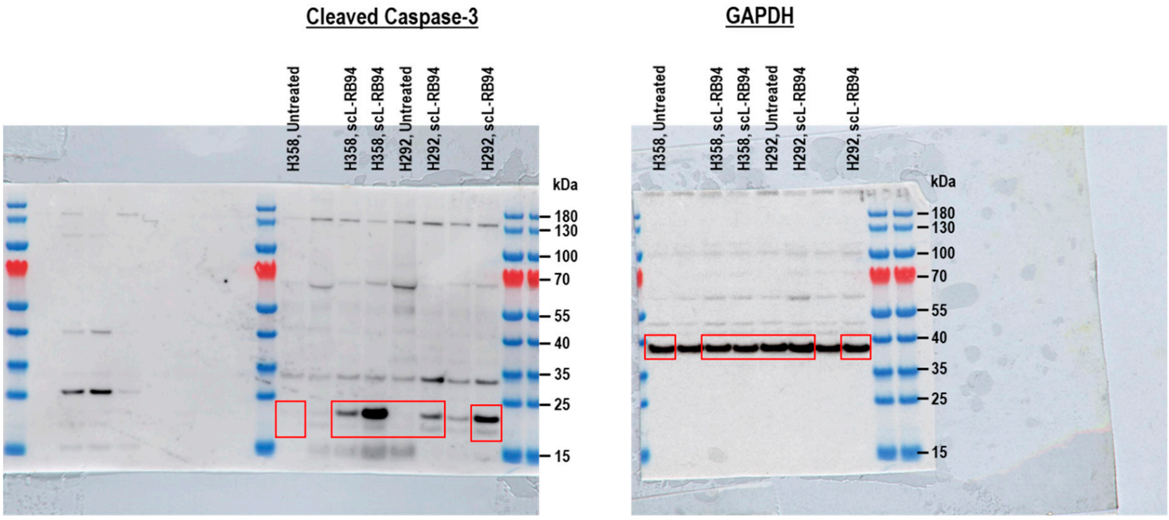

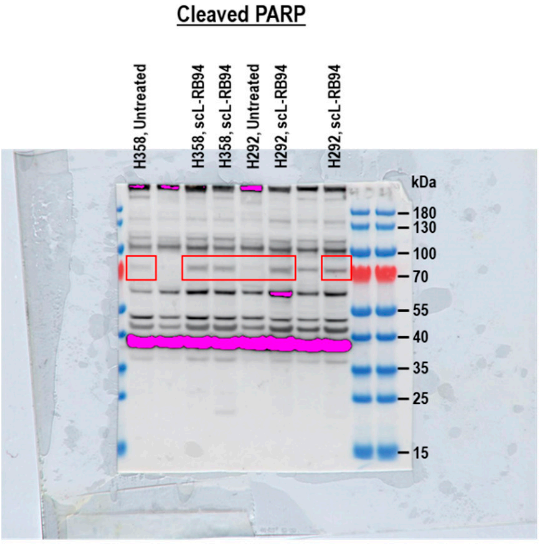

Target	Sample ID	Pixel density
cCasp3	H358, Untreated	1663
	H358, scL-RB94	12676
	H358, scL-RB94	35916
	H292, Untreated	5644
	H292, scL-RB94	12791
	H292, scL-RB94	23157
cPARP	H358, Untreated	4305
	H358, scL-RB94	11898
	H358, scL-RB94	11147
	H292, Untreated	4068
	H292, scL-RB94	18882
	H292, scL-RB94	17454
GAPDH	H358, Untreated	20734
	H358, scL-RB94	20745
	H358, scL-RB94	20662
	H292, Untreated	23490
	H292, scL-RB94	25616
	H292, scL-RB94	22003

Figure S2. Original whole Western blot Images for Figure 1F. The densitometry readings for the western bands are shown in table.

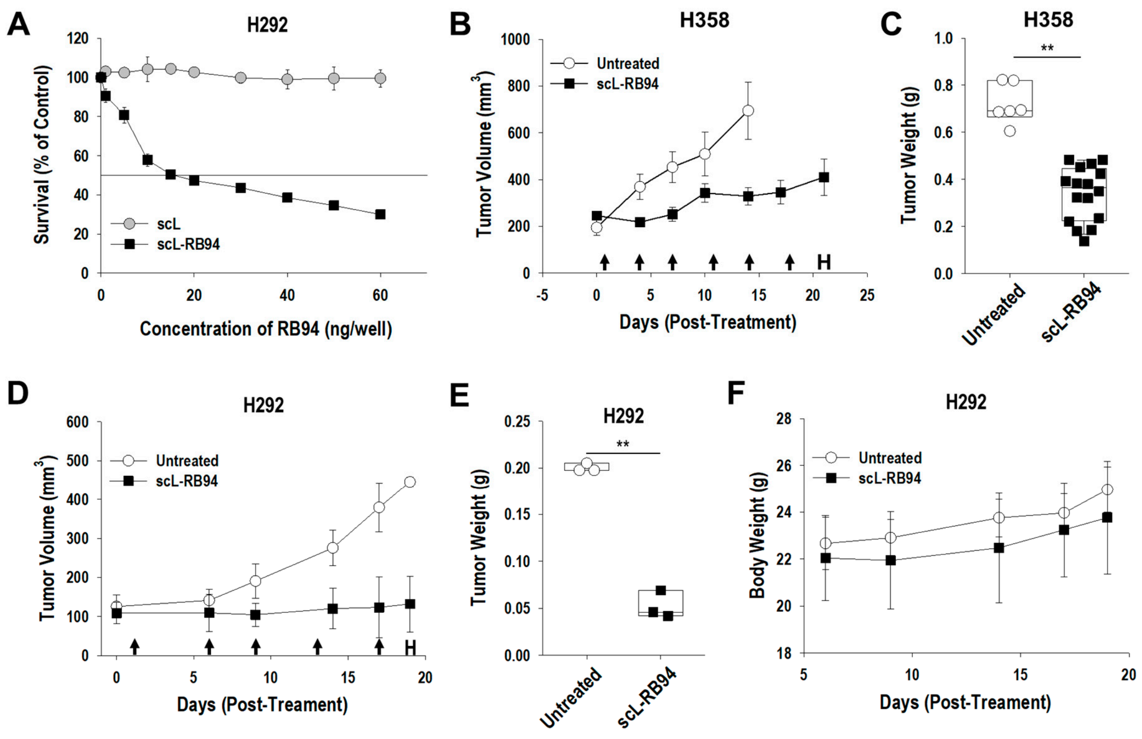

Figure S3. scL-RB94 effectively kills human NSCLCs and suppresses tumor growth. (**A**) H292 was transfected with increasing concentrations of scL-RB94 and cell viability was measured by XTT assay 72h later. For comparison, cells were also treated with an empty nanocomplex without payload (scL). (**B**) Athymic mice bearing subcutaneous H358 tumor received total 6 injections (30 µg DNA/injection/mouse) of scL-RB94 over 18 days as indicated by arrows (N=6-16). Tumor growth was monitored by measuring tumor volumes. (**C**) H358 tumors were harvested and weighed on day 21 (indicated by H). **p<0.001, Student's t test. (**D**) Athymic mice bearing subcutaneous H292 tumor received total 5 injections (30 µg DNA/injection/mouse) of scL-RB94 over 17 days as indicated by arrows (N=5). Tumor growth was monitored by measuring tumor volumes. (**E**) H292 tumors were harvested and weighed on day 19 (indicated by H). **p<0.001, Student's t test. (**F**) Body weight change of H292 tumor bearing mice receiving scL-RB94.

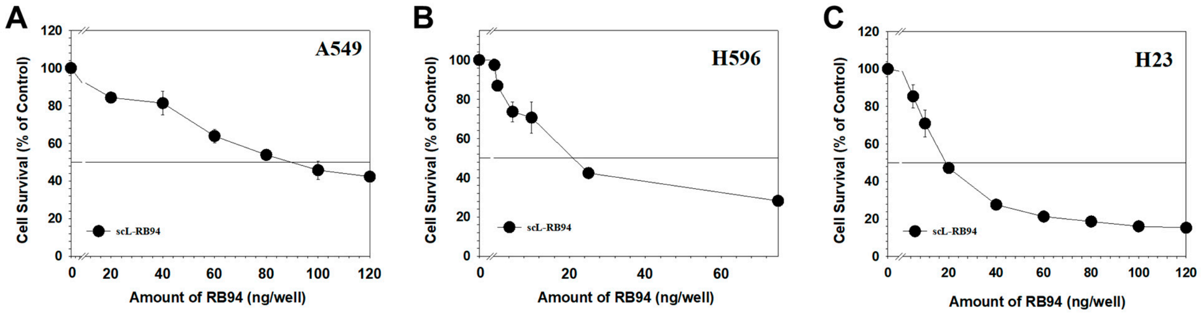

Figure S4. scL-RB94 effectively kills human NSCLCs *in vitro*. Cell viability was measured by XTT assay 72h after transfection with increasing concentrations of scL-RB94 in (**A**) A549, (**B**) H596, and (**C**) H23 cells.

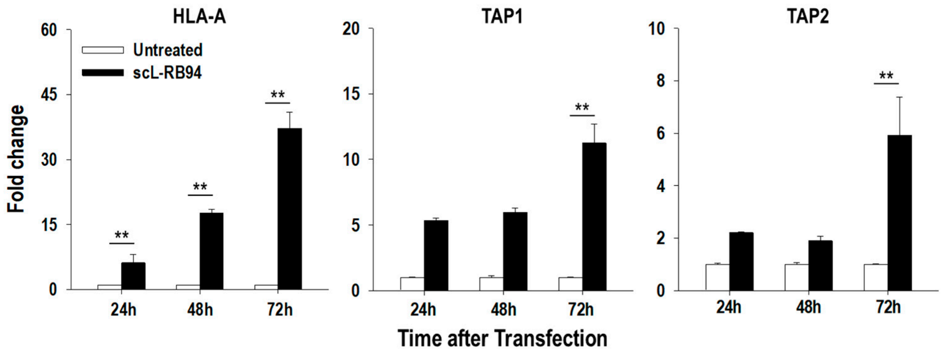

Figure S5. Quantitative RT-PCR analysis. mRNA level of antigen presentation molecule, HLA-A, and antigen processing molecules, TAP1 and TAP2 in H292 cells after treatment with scL-RB94 *in vitro.* **p<0.001, Student's t test.

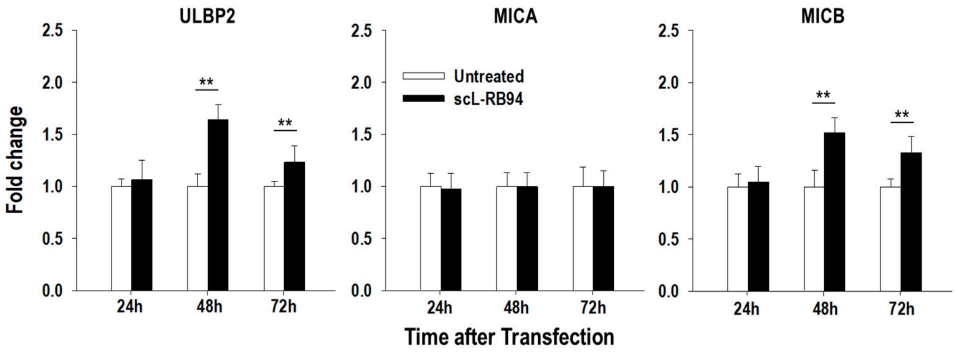

Figure S6. Quantitative RT-PCR analysis. mRNA level of ligands (ULPB2, MICA, and MICB) associated in NK cell activation in H358 cells after treatment with scL-RB94 *in vitro.* **p<0.001, Student's t test.

Table S1. Human NSCLC lines tested in the current study. The status of the oncogenes and tumor suppressor genes was gathered from the Sanger Institute COSMIC database (https://cancer.sanger.ac.uk/cosmic).

Cell Line	Type	RB	TP53	KRAS	CDKN2A	EGFR	RB94 IC_{50} (ng/well)
NCI-H358	bronchioalveolar carcinoma	wt	wt	mt	wt	wt	24.1
NCI-H292	mucoepidermoid pulmonary carcinoma	wt	wt	wt (▽)	wt (△)	wt	15.7
A549	carcinoma	wt	wt	mt	wt (⬇)	wt	84.2
NCI-H596	adenosquamous carcinoma	mt	mt	wt (▽)	wt	wt (△)	19.4
NCI-H23	adenocarcinoma	wt	mt	mt (△)	wt	wt	18.5

mt = mutated, wt = wild-type, ⬇= loss in copy number,△= over-expressed,▽= under-expressed

Table S2. The size and zeta potential of nanocomplexes. scL nanocomplexes encapsulating various payloads as determined by the Zetasizer immediately after the complexes were freshly prepared. Units for number, volume, and intensity are in (d.nm). Units for zeta potential are in (mV).

Nanocomplex	PDI	Number (d.nm)	Volume (d.nm)	Intensity (d.nm)	Zeta potential (mV)
scL	0.26 ± 0.01	28.56 ± 1.89	45.53 ± 5.94	105.11 ± 17.34	56.09 ± 1.63
scL-vec	0.19 ± 0.04	92.25 ± 26.74	177.06 ± 11.24	197.07 ± 3.33	31.35 ± 2.27
scL-RB94	0.20 ± 0.02	96.41 ± 23.39	171.14 ± 27.14	195.23 ± 12.50	27.49 ± 0.76
